# Tracing Jomon and Yayoi ancestries in Japan using ALDH2 and JC virus genotype distributions

**DOI:** 10.1186/s13323-015-0031-1

**Published:** 2015-12-30

**Authors:** Daisuke Miyamori, Noboru Ishikawa, Nozomi Idota, Yasuhiro Kakiuchi, Stuart McLean, Tadaichi Kitamura, Hiroshi Ikegaya

**Affiliations:** Department of Forensic Medicine, Graduate School of Medical Science, Kyoto Prefectural University of Medicine, 465 Kajiicho, Kamigyo, Kyoto, 602-8566 Japan; Asoka Hospital, 1-8-1, Sumiyoshi, Koto, Tokyo, 135-0002 Japan

**Keywords:** JC virus, ALDH2 mutation, Japanese

## Abstract

**Background:**

According to the *dual structure model*, the modern Japanese ethnic population consists of a mixture of the Jomon people, who have existed in Japan since at least the New Stone Age, and the Yayoi people, who migrated to western Japan from China around the year 300 bc Some reports show that the Yayoi are linked to a mutation of the aldehyde dehydrogenase 2 gene (ALDH2).

Recent viral studies indicate two major groups found in the Japanese population: a group with the CY genotype JC virus (JCV) and a group with the MY genotype JCV. It is unclear whether either genotype of the JC virus is related to the Jomon or Yayoi.

In this study, we attempted to detect JCV genotypes and ALDH2 mutations from the DNA of 247 Japanese urine samples to clarify the relationship between the dual structure model and the JCV genotype through ALDH2 mutation analysis and JCV genotyping.

**Findings:**

The ALDH2 polymorphism among 66 JC virus-positive samples was analyzed, and it was found that the ALDH2 variant is significantly higher in the population with CY genotype JCV (51.5 %) than in the population with the MY genotype (24.2 %) (*p* < 0.05).

**Conclusion:**

From these findings, it may be inferred that the ALDH2 mutation, which is related to the Yayoi, is related to CY genotype JCV. When the Yayoi migrated to the Japanese archipelago, they brought the ALDH2 mutation as well as the CY genotype JCV.

## Introduction

**Fig. 1:**
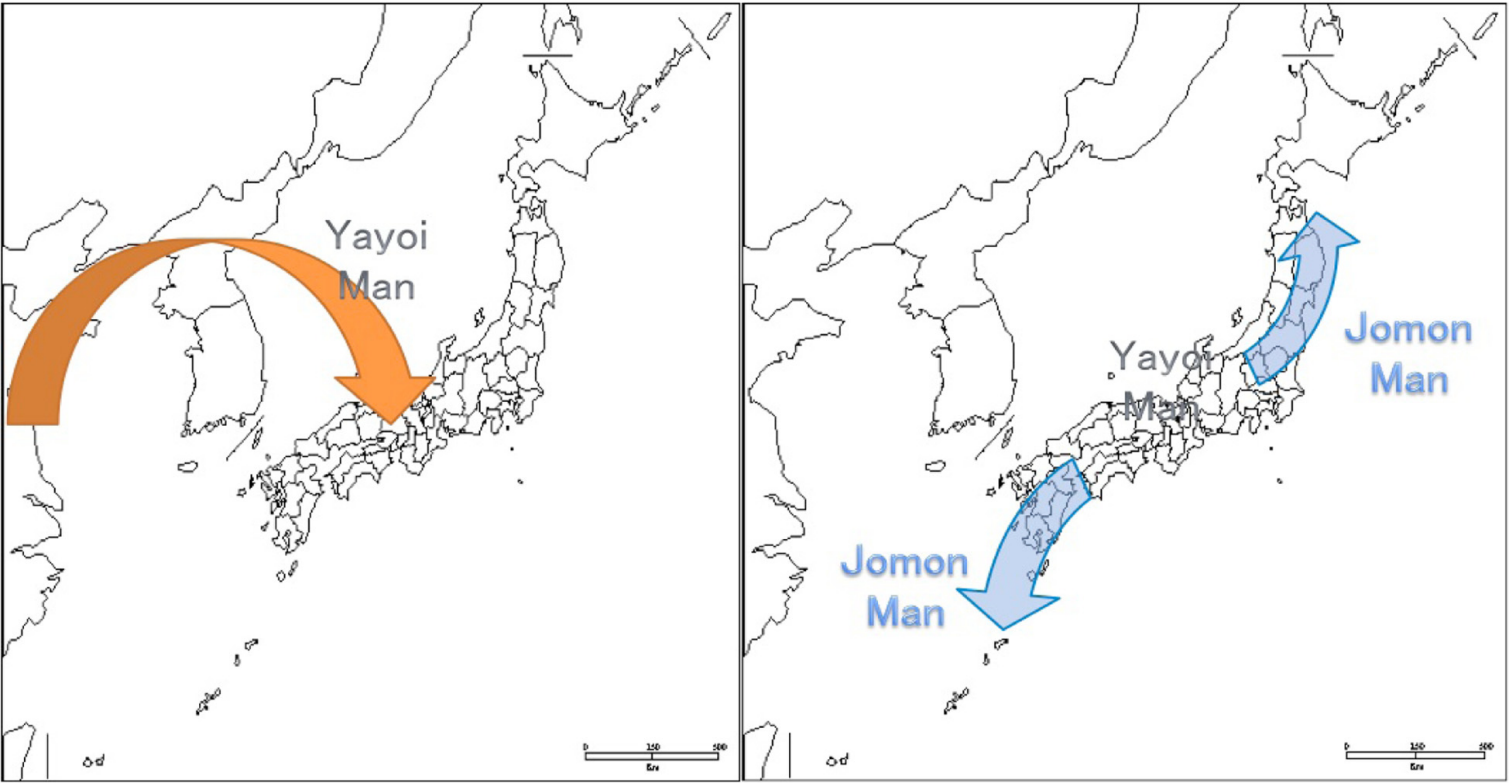
This map shows the migration of the Yayoi people and the Jomon people. The Jomon people moved to the north and south of Japan after the Yayoi people migrated to central Japan

It is believed that the modern Japanese ethnic population consists of a mixture of the Jomon people, who have existed in Japan since at least the New Stone Age, and the Yayoi people, who migrated to western Japan from China via Korea approximately 2000 to 3000 years ago and were responsible for spreading rice cultivation [25] (Fig. 1). There are many studies that describe this *dual structure model* of the Japanese people based on analysis of elements such as the human Y chromosome, mitochondria, single nucleotide polymorphism (SNP), archeological data and historical records [3, 7–10, 19–21, 26, 28]. Sokal and Thompson [26] interpreted the cause of the genetic distribution to have resulted from the selection, admixture and resolving power of a locus that reflects a particular population event. The aldehyde dehydrogenase 2 gene (ALDH2) is one of the examples among studies.

Previous studies have shown that approximately 2000 to 3000 years ago, a variant of ALDH2 was present within the Chinese population. Citing historical evidence [18], researchers speculated that people with this ALDH2 variant migrated to western Japan 2000 to 3000 years ago. Oota et al. [22] claimed that based on actual DNA evidence, this ALDH2 variant was a young haplotype associated with low levels of genetic diversity. Therefore, it may be possible that this mutation of the ALDH2 gene and the Yayoi migration from China to Japan happened in roughly the same period.

**Fig. 2:**
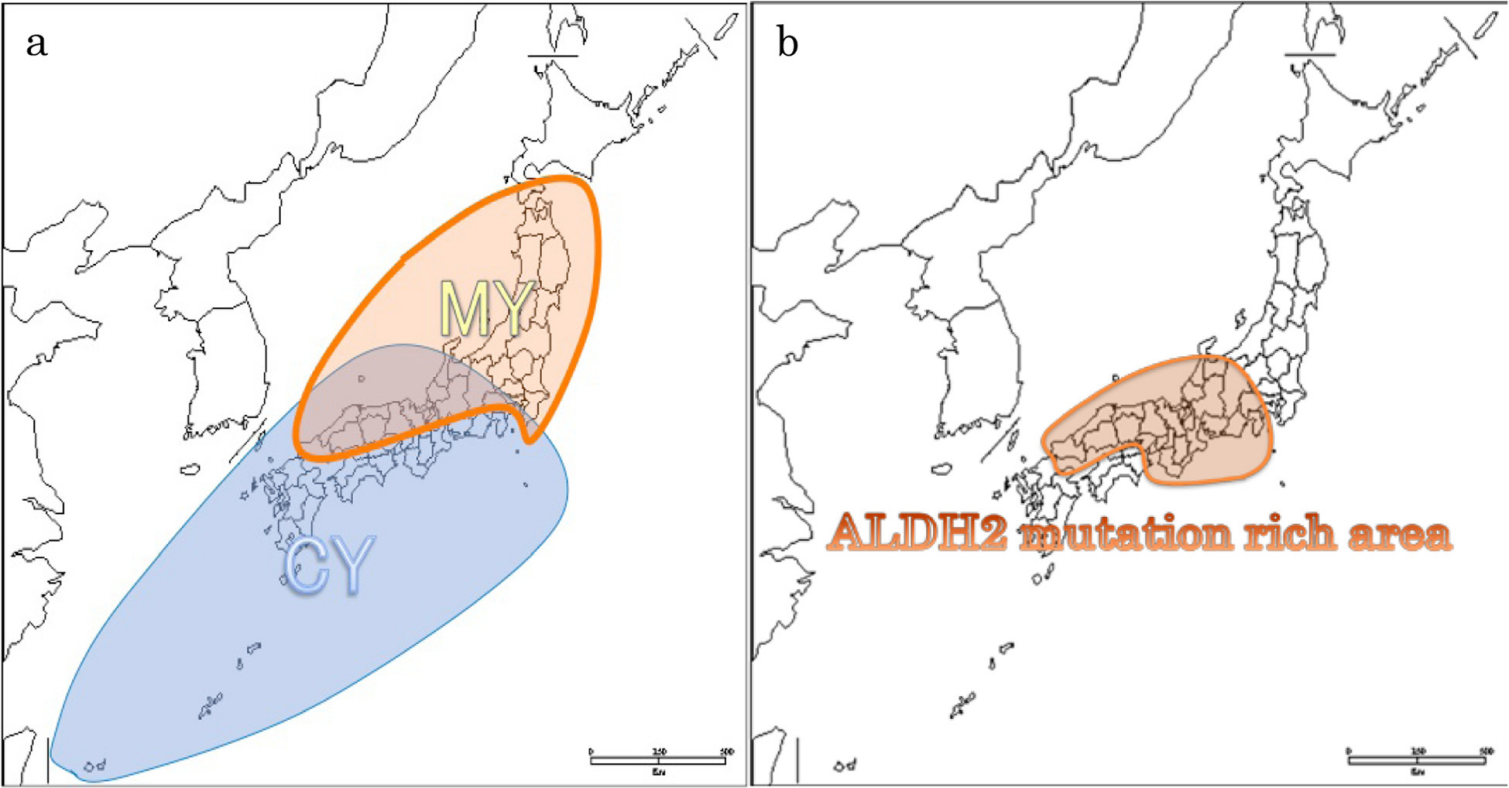
**a** Map showing the distribution of the two major JCV genotypes (CY and MY) in Japan. The areas in which CY and MY were found more frequently (i.e., at rates >75 %) are indicated as CY-rich or MY-rich areas, respectively. The area designated as the intermediate area is where the genotypes CY and MY were found at almost identical frequencies. This map was created utilizing data reported by Kitamura et al. [14]. **b** The geographic distribution of the ALDH2 variant. The orange area indicates where the frequency of the ALDH2 variant is over 24 % of the population. The ALDH2 mutation-rich area is central Japan. This map was created utilizing data reported by Li et al. [17]

The JC virus (JCV), one of the *polyomaviruses*, is a useful marker in tracing the dispersal of human populations [30]. This is because once an individual is infected asymptomatically with JCV during childhood [23, 24], the initially infected JCV strain persists in renal tissue for life, and other strains of JCV are unable to infect the already infected individual [2, 13, 29]. There are more than 20 main JCV genotypes that are distributed in geographically distinct domains throughout the world [30]. Two major types of JCV genotypes, CY and MY, are found in the Japanese archipelago [14] (Fig. 2a). The genotype CY is commonly distributed in western Japan, northeast China, and Korea [5, 6] and is not found in other places [27, 32]. Earlier studies suggested that the Chinese might have brought the CY genotype JCV to Japan when they migrated from China [14]. The genotype MY is commonly distributed in eastern Japan and among Native Americans [27, 32].

This JCV distribution may support the dual structure model of the Japanese population. However, there have been no studies verifying the correlation between the distribution of the CY genotype JCV and the dual structure model.

Therefore, through the detection of the ALDH2 mutation among JCV-positive Japanese samples, we investigated its correlation with the CY genotype JCV, and we discuss whether the dual structure model is also supported by JCV genotype in this study.

## Materials and methods

### Materials

In this study, 50-mL urine samples were donated under informed consent by 247 healthy volunteers who live in the Japanese cities of Hirosaki, Hachinohe, Kazuno, Akita, Omagari, Koromogawa, Yonezawa, Nara, Kaizuka, and Matsue. Extracted DNA samples from the urine were analyzed. The DNA extraction method used for the samples was as described by Kato et al. [12].

This research work was approved by the institutional review board of the Kyoto Prefectural University of Medicine (G-112).

### JCV genotype classification

The 610-bp IG region [1] that encompasses the 3′-terminal regions of both T antigen and VP1 genes was PCR-amplified using primers P1 and P2, and ExTaq^EM^ polymerase (TaKaRa bio Inc., Tokyo, Japan), as described by Kunitake et al. [16].

The IG region was previously established as a region of the JCV genome that contains abundant type-determining sites. Utilizing the RFLP (restriction fragment length polymorphism) technique, the JCV genotype from the urine samples was analyzed.

The restriction enzymes NlaIII, DdeI, Hinf I, and PvuII (BioLabs, MA) were utilized. Using standard protocol, a 25-μL aliquot of a purified PCR mixture was digested at 37 °C for 1 h with 10–20 units of restriction enzyme. The digestion was resolved by electrophoresis on a 2.0 % agarose gel, and genotype was determined as described by Kitamura et al. [14].

### ALDH2 genotype classification

The mutation of Glu504 Lys of the ALDH2 gene was detected and classified among the JCV-positive samples utilizing real time-PCR. Fluorescence melting curve analysis was performed using a LightCycler (Roche Diagnostics GmbH, Mannheim, Germany) with primers and probes obtained from Takara Bio Inc. (TaKaRa Cycleave Human ALDH2 Typing Probe/Primer Set).

PCR conditions were as follows: initially at 95 °C for 10 s, followed by 60 cycles of thermal shift at 95 °C for 5 s, annealing at 53 °C for 10 s, and extended at 72 °C for 20 s. The fluorescence emitted was measured during this process.

### Statistical analysis

The chi-square independence test was utilized to compare the presence of the ALDH2 variant in samples with the JCV genotype MY and in samples with the JCV genotype CY. Additionally, the chi-square independence test with Yates’ correction was utilized to compare the presence of wild-type ALDH2, (i.e., ALDH2 gene has normal activity) in samples with the JCV genotype MY and in samples with the JCV genotype CY. The association between ALDH2 genotypes and JCV genotypes was expressed in terms of the *p* value. The significance was set as 5 %. All analyses were performed using Microsoft Excel software.

## Results

JCV was detected in 66 samples. Among them, the genotype CY was detected in 33 samples and the genotype MY was detected in 33 samples. Among the 33 CY detected samples, the ALDH2 variant was detected in 17 samples (51.5 %). Among the 33 MY detected samples, the ALDH2 variant was detected in only 8 samples (24.2 %). All of the ALDH2 variants were Glu/Lys heterozygotes. Lys/Lys homozygotes were not detected in the samples. The ALDH2 variant was found more commonly in people who carry the CY genotype JCV than in people who carry the MY genotype JCV (Table 1) (*p* value 0.04, odds ratio 0.30).

**Table 1:** Association between the JCV genotype and the ALDH2 variant

	MY genotype	CY genotype
ALDH2 variant	8	17
(Glu/Lys heterozygote)		
ALDH2 wild type	25	16
(Glu/Glu homozygote)		
Total	33	33

Lys/Lys homozygote was not detected in this study.

## Discussion

The results of the present study suggest that the ALDH2 variants are more prevalent in people with CY genotype JCV than in people with MY genotype JCV (Table 1).

In an earlier study, the CY genotype JCV was found to be more common in western Japan, and the MY genotype JCV was found to be more common in Eastern Japan [14] (Fig. 2a). The CY genotype JCV was also found in China [5, 6], but the MY genotype JCV was found mainly in the Japanese archipelago and in North and South America [32]. Estimated from the substitution rate of the JCV genome, the MY clade occurred more than approximately 10,000 to 30,000 years ago [31], and the CY clade occurred approximately 10,000 years ago. Therefore, the MY genotype JCV initially occurred in the Japanese archipelago and spread to the Americas, and later, the CY genotype JCV migrated from China to Japan. It is possible that the ALDH2 mutation only occurred within people who carry the CY genotype JCV, explaining why it is uncommon for people who carry the MY genotype JCV to have the ALDH2 mutation. Another study indicates that Native Americans do not have the ALDH2 mutation [4]. Combining this information with the results of Zheng et al. [32], we can speculate that people who carry the MY genotype JCV may not have originally had the ALDH2 mutation. Those findings also support the relation of the CY genotype JCV with the ALDH2 mutation. Therefore, it is inferred that the ALDH2 gene mutation spread into East Asia in the past few thousand years. This may be a good example of a locus subjected to selection, displaying wide distribution, and high frequency with low associated variation, confined to a continental region.

However, there are some people with the ALDH2 mutation and the MY genotype JCV, and some people are without the ALDH2 mutation and with the CY genotype JCV.

It is believed that there are several reasons for this. First, extensive genetic mixing between Yayoi and Jomon is expected to have occurred after the major migration 2000 to 3000 years ago and before the present day, when our samples were collected. Second, the ALDH2 genes from both parents are passed down and combined forming their child’s ALDH2 gene type. As a result, there is a 50 % possibility of inheriting the ALDH2 mutation from a father or mother carrying the gene. However, in the case of the JCV genotype, the possibility of infection with JCV from one’s father or mother is affected by the number of exposures to their urine. As a result, if the infection rate of one JCV genotype is high within a given area, subsequent generations are less likely to be infected by JCV of other genotypes, which in turn becomes less common through natural selection. Minority groups of viral genotypes are excluded by the dominant genotype within a given area during a given period. These differences in the acquisition of infection from a local majority or minority strain of a virus or the inheritance of a mutant or non-mutant version of a gene on the other might be the cause of the differences in distribution of the JCV genotypes and the ALDH2 mutation in Japan (Fig. 2a, b).

After a study reported evidence of greater genetic affinity between Ainu and Ryukyuan people (i.e., people indigenous to Okinawa and a surrounding chain of islands between Japan and Taiwan) than between either group from the Japanese mainland populations [11], it was suggested that the Jomon migrated to the north and south of the Japanese archipelago in response to the Yayoi migration from China to western Japan (Fig. 1). Because the ALDH2 mutation is thought to be common among Yayoi, the distribution of the CY genotype JCV is believed to be the same as the distribution of those people who have the ALDH2 mutation (Fig. 2a, b). However, Kitamura et al. [14] reported that the most common genotype found in Okinawa is CY. This is contradictory to the idea of the Jomon migration to Okinawa and Tohoku (the northeast region of the main island of Japan) with their MY genotype JCV, following the migration of the Yayoi people to the main islands of the Japanese archipelago. According to our model, all specimens from before the Yayoi arrival would lack both the ALDH2 mutation and the CY viral genotype while bearing the MY genotype. Further studies are necessary to examine other aspects of the Yayoi arrival. The potential value of studying ancient DNA in pre-Yayoi era specimens of the Ryukyuans and other populations in Japan using viral genome capture techniques and ALDH2 mutation analysis could help solve the mystery of the apparent rarity of the MY variant JCV in modern Ryukyuan populations [15].

## Conclusion

From these findings, it may be inferred that the ALDH2 mutation, which is related to the Yayoi, is related to CY genotype JCV. When the Yayoi migrated to the Japanese archipelago, they brought the ALDH2 mutation as well as the CY genotype JCV.

## Abbreviations

ALDH2: aldehyde dehydrogenase 2
BKV: BK virus
JCV: JC virus
PCR: polymerase chain reaction
RFLP: restriction fragment length polymorphism
SNP: single nucleotide polymorphism
